# Tissue Banking, Bioinformatics, and Electronic Medical Records: The Front-End Requirements for Personalized Medicine

**DOI:** 10.1155/2013/368751

**Published:** 2013-05-30

**Authors:** K. Stephen Suh, Sreeja Sarojini, Maher Youssif, Kip Nalley, Natasha Milinovikj, Fathi Elloumi, Steven Russell, Andrew Pecora, Elyssa Schecter, Andre Goy

**Affiliations:** ^1^The Genomics and Biomarkers Program, The John Theurer Cancer Center at Hackensack, University Medical Center, D. Jurist Research Building, 40 Prospect Avenue, Hackensack, NJ 07601, USA; ^2^Sophic Systems Alliance Inc., 20271 Goldenrod Lane, Germantown, MD 20876, USA; ^3^Siemens Corporate Research, IT Platforms, Princeton, NJ 08540, USA; ^4^Research for the Cure Foundation, Hillsdale, NJ 07642, USA

## Abstract

Personalized medicine promises patient-tailored treatments that enhance patient care and decrease overall treatment costs by focusing on genetics and “-omics” data obtained from patient biospecimens and records to guide therapy choices that generate good clinical outcomes. The approach relies on diagnostic and prognostic use of novel biomarkers discovered through combinations of tissue banking, bioinformatics, and electronic medical records (EMRs). The analytical power of bioinformatic platforms combined with patient clinical data from EMRs can reveal potential biomarkers and clinical phenotypes that allow researchers to develop experimental strategies using selected patient biospecimens stored in tissue banks. For cancer, high-quality biospecimens collected at diagnosis, first relapse, and various treatment stages provide crucial resources for study designs. To enlarge biospecimen collections, patient education regarding the value of specimen donation is vital. One approach for increasing consent is to offer publically available illustrations and game-like engagements demonstrating how wider sample availability facilitates development of novel therapies. The critical value of tissue bank samples, bioinformatics, and EMR in the early stages of the biomarker discovery process for personalized medicine is often overlooked. The data obtained also require cross-disciplinary collaborations to translate experimental results into clinical practice and diagnostic and prognostic use in personalized medicine.

## 1. Introduction

Research in personalized medicine seeks to achieve optimal clinical outcomes through the use of innovative biomarker discoveries to develop drugs that best suit a specific group of patients. To derive best-fit treatment options for a specific patient group, various signaling pathways are thoroughly analyzed to identify altered molecular circuitry that initiates and maintains the clinical phenotype of the disease. For cancer, this altered signaling promotes a cascade of molecular events, that is, cell- or tissue type-dependent, and this relationship gives rise to a specific biomarker set that has a direct association with the cancer phenotype. The unique genetic profile of an individual patient's cancer will generate specific gene expression signatures and modifications of genes/miRNA, proteins, and metabolites. 

Current and future technologies will produce a flood of data, not only from the laboratory bench but also from clinical sources, and the association of these data with a specific cancer phenotype is expected to be more sensitive and to have a higher specificity that will allow increasingly accurate clinical decision making. Mutations are often associated with specific cancer malignancies, and the combination of well-documented mutations with better-fit drugs has the potential to provide a good clinical outcome with fewer side effects for patients. For example, Cyp2D6 genotyping at the time of breast cancer diagnosis provides prognostic information regarding the effectiveness of tamoxifen treatment [1]. If a poor clinical outcome is predicted, then additional data and evidence-driven clinical decisions may allow better alternative therapy options for those patients. With recent advances, the ER, PR, and Her2 status of breast cancer patients can be used to make therapeutic choices that include a customized tamoxifen regimen to prevent disease recurrence [2]. The next generation of sequencing and “-omics” technologies will continue to improve our ability to recognize cancers, improve treatments, and track the health of survivors [3]. Scientific data from “-omics” now represent many pathways and molecular signatures that are directly involved in triggering cancers, and these research data can be applied to diagnosis, prognosis, and treatment decisions [4].

As the field of personalized medicine evolves, scientific and clinical data are expected to converge in a common central database, which will facilitate the exchange of data that can be transformed into valuable information that will aid clinical decisions, enable customized treatments, and generate new advances for cancer medicine. These developments would be cost effective for both patients and health care practitioners because a proper course of action could be identified using the stored data, which will help avoid the use of unnecessary tests and ineffective therapies. With the implementation of the Affordable Care Act “Obamacare” in 2014, economical assays that provide accurate results will be needed to determine the most effective treatments and ensure the best outcomes. In evaluating the cost of screening, assay costs and the cost effectiveness of drugs must be considered and should correlate with the cost of biomarker detection in patient samples [5]. Screening assays will provide tools for making the most accurate clinical decisions, such that the customized treatment represents a best fit, if not a match, and targets molecular changes that occur in the patient's tumor. Companion assays for each targeted drug will maximize the treatment benefit options for a specific cancer. Minimally invasive procedures for obtaining small biospecimens from patients will also help drive down medical costs to satisfy the new regulations. The combined scientific and clinical information from each cancer patient can be further analyzed and compared precisely with traditional treatments, and any new treatments with companion diagnostics can be tested in clinical trials. Thus, elucidating the molecular profile of each patient involves testing for robust biomarkers with high sensitivity and specificity for particular cancer phenotypes and requires high-quality biospecimens and well-organized tissue banking. Scientific information must be integrated with clinical information derived from well-documented electronic medical records (EMRs) using informatics tools, although to date the importance of this integration has been largely neglected by the research and clinical community. In combination, tissue banking, bioinformatics, and EMRs are front-end requirements for enhancing personalized medicine, and they represent key resources that will determine the path of progress in translational research to support clinical applications. 

Bioinformatics encompasses a combination of statistics, molecular biology, and computer science to store and analyze biological data, while tissue banking involves procurement of tissue or tumor samples during medical procedures and their collection and storage in a tissue repository. By integrating patient information and experimental results, novel biomarkers can be discovered that may have a major effect on the way patients receive care and could also greatly influence drug and diagnostic customization [6]. Biomarkers can be proteins, genes, metabolites, or even methylation patterns that are used to detect genetic tendencies for specific types of cancers and other diseases (Table 1) [7]. Depending on the cancer, certain diagnostic tests are performed, and if a given biomarker is found in a patient sample, it will guide the choice of treatment. In the case of melanoma, for example, a BRAF mutation test is used to determine if a BRAF V600E mutation exists in the patient sample, and if present, that patient will receive a customized treatment with the drug Zelboraf, which is effective only for BRAFV600E-positive melanomas. Nonsmall cell lung cancer (NSCLC) patients carrying an ALK mutation are treated with the drug Xalkori, which is specific for this type of mutation, whereas NSCLC patients having an EGFR mutation would receive alternative customized treatments. 

These well-studied biomarkers enhance the ability of clinicians to determine a course of treatment with minimal toxicity. However, there remains a need for technologies to assist with the discovery and transformation of biomarkers to diagnostic uses [7]. One approach is the use of aptamers, small DNA/RNA oligonucleotides having high selectivity for their target, which can help avoid the limitations imposed by antibodies in diagnostic applications [8]. To initially identify potential cancer-specific biomarkers, biomarker discovery applications require bioinformatics data, information from which can then be used for analysis of samples stored in tissue banks. Sample quality and the associated clinical information are also important factors in biomarker discovery because data from all components are required to complete this process [9, 10]. Table 1 includes a list of currently available drugs in cancer therapy, developed after identifying relevant biomarkers and companion diagnostics [11–41].

Given the variety of data and samples being collected and advances in personalized medicine, the importance of collaborations between scientists and clinicians, as well as industry and academia, becomes evident. Targeted therapies, therapeutic resistance, challenges of “-omics” technologies, and even issues with biomarker-related trials can be approached only through collaboration [42]. There are scientific uncertainties present in all areas under study, which medical research alone cannot address. However, through a transdisciplinary scientific methodology, these disparate areas can be combined to offer innovative and unique diagnostic and treatment plans [6]. 

## 2. Importance of Tissue Banking

In the past two decades, efforts to create and maintain tissue banks have provided a foundation for future research toward personalized medicine in cancer. After potential biomarkers have been identified through the use of bioinformatics and an experimental design has been put into place, tissue banks come into play. If, for example, an institution wants to examine biomarkers associated with ovarian cancer, a query of the records of the tissue bank will return sample catalog numbers pertaining to ovarian cancer enabling experiments to commence. The most important and potentially problematic aspect of a successful tissue bank is the quality of stored samples. Proper procurement methods, sample handling, and the bank itself are crucial since biomarker investigations performed using low-quality samples will likely generate erroneous and misleading data [43]. 

Additionally, relatively few patients consent to have their biosamples banked, possibly due to a lack of understanding regarding the use of their tissue and the concern that the information gained could be used against them in the future. Therefore, to improve the rate of consent, improved education of the public and patient populations regarding the safety and value of such donations is needed. In addition, the consent process, which involves long, difficult to understand forms that must be read and understood, could be made less burdensome. One approach to improve education would be to employ models for software engagement that have been successful in commercial and educational settings. These include 3D immersive settings that emulate real-world situations, such as in the Second Life virtual world platform, where dynamic choices and outcomes can result in good or bad medical outcomes [44]. Some features of multiuser games would likely be of sufficient interest to sustain the attention of a variety of potential donors and increase patient awareness of tissue donation. Constructing and deploying these sorts of educational tools in public and medical venues, such as in malls and doctors' offices, or in free apps for smart phones or tablets could be of value in informing patients of the importance of tissue banking as applied to research discoveries and treatment successes. To increase the number and variety of biosamples, new approaches can be applied for patient education and involvement. One promising approach for increasing the rates of consent to donate biospecimens was inspired by modern online software that encourages interactive learning for casual users [45]. Such game-like interfaces were shown to be useful in medical education and training [46]. Better-informed patients who consent more often will enrich the number and variety of samples in the tissue banks, which will support the data analysis and integrated information bases that can lead to discoveries and treatments. An example session might involve an individual interacting with a public kiosk that allows some user input and provides an immersive environment where the user engages another character who plays the role of a doctor asking for consent to use a tissue sample for research purposes. During the game-like session, the user could be led through a set of choices based on their actions and responses in realistic scenarios. The encounters and selection sequence could be engineered such that consent form issues are clarified. Complex issues such as biomolecular interactions and genetic conditions could be illustrated in attractive 3D renderings, so as to better visualize biological systems and to focus more precisely on why certain therapies are suggested. During the session, the importance of consenting to donate biospecimens could be reinforced and the user rewarded by illustrations of improved health of other patients based on more complete information gleaned in part from donated tissue samples. The play could also educate the potential donor in how such general consents could eventually be of personal benefit in the event of negative health conditions later in their life.

While tissue banks archive and provide biospecimens for translational research and healthcare innovations, there are challenges to be faced before sample procurement from donors. Obtaining informed donor consent is a major consideration, as the health care system considers informed consent to be legally and ethically required to ensure donor data security. Moreover, informed consent is an autonomous act, and by signing the consent, the patient/donor confirms that he/she understands the risks and benefits of research, is protected from exploitation, and learns the purpose of the research for which the donations are made [47]. Recent research by Beskow et al. suggests that combining a simplified consent form with supplemental materials aimed at patients who wish to have additional information about the research would minimize the complications in understanding research goals [48]. Similarly, there are opt-in and opt-out criteria for participating in specified research. Opt-out methods are “passive” allowing the participants/donors to decide whether to be excluded from the research. Often, the opt-out approach provides little information to the donor regarding the exact research goals. Opt-out also provides some choices for participants/donors within a limited timeframe, which helps to procure more samples to accelerate research. The opt-in method signals the willingness of participants/donors to take a more active role in the research; this method receives more public acceptance with more information and participant education [47]. The broad consent given by a participant permits use of their biospecimens and personal medical information for future unspecified research under appropriate IRB regulations. Broad consent is more of a “general” criterion, which allows the donor to be informed about the various processes involved in tissue bank functions but with limited or no information about the nature of future research. Broad consent has advantages over specified informed consent because it allows biospecimens to be used for diverse research activities and minimizes the practical difficulties in transporting consent to the context of tissue banks. 

Patients are more likely to give consent if they have been educated with respect to the benefits of tissue banking prior to the hospital visit; thus, public awareness plays a vital role in encouraging tissue donation [49]. Efforts from community advocates and patient advocate groups can make the consenting process “personalized” as well. This can be accomplished by individuals that represent patients/donors and their families, who have already consented and donated biospecimens. These “alumni” groups or individuals can speak in family gatherings or community events and clarify the confusion and fear that may accompany the cancer diagnosis and anticipated medical procedures. In the absence of reliable, accurate information, misconceptions about additional procedures, treatments, and tests can deter a patient from making a well-informed decision. Conversations—without the confusing medical jargon—with friends and community members who have gone through the experience can help potential patients/donors and their families understand the importance of donation for tissue banking. A small, friendly conversation with the “person next door” atmosphere can make it personal enough for the patient/donor to give consent for tissue banking. This type of personal conversation can be adopted for a national stage at patient advocate conferences or convention. The fact that a specific medical condition becomes a top search query on the internet on the same day it is described by the national media illustrates the eagerness of general public and communities to learn when information is distributed appropriately. We expect similar responses as previously mentioned when efforts are provided to the general public and patient communities regarding the importance of tissue banking and educate about the role of tissue banking for innovations related to personalized medicine. 

Information technology plays a major role in management of biobanks. Biospecimen information and corresponding molecular and clinicopathological data is currently stored primarily using XML for web-based exchange of information. This system consists of different servers including web, application, database, authentication, and authorization [50]. The system connects with other relevant databases within a hospital enabling storage and retrieval of clinical, pathological, and personal data with highly secure access. Ensuring security for accessing records is provided via digital codes or smart cards for users. The system can be customized depending upon the requirements of the biobank [50]. Software interfaces play a role in storage and retrieval of larger data sets associated with biospecimens detailing storage location, history, and storage time providing easy traceability of samples. The Laboratory Information Management System (LIMS) is a flexible, expandable, and secure software interface used for storing and retrieving large amounts of data generated through the processing of biospecimens in research labs [51]. For example, screenings for genetic mutations in an automated environment require data storage, retrieval, and tracking. LIMS helps minimize human errors in data processing by communicating with lab equipment, robots, and databases and enables easy retrieval of data [51]. 

The implementation of BIMS (Biobank Information Management System) ensures the integration of data from different sources, such as various research institutions, which may employ different formats and procedures. BIMS resolves data integration issues in biobank research through a series of sequential processes. Extraction, de-identification, consolidation, abstraction, and query [52] are individual components that handle and manage data. The extraction component receives data from external sources, which are continuously updated and/or are in different formats depending on the system used to generate the data (e.g., text files, spread sheets). Deidentification attaches a unique identifier to each data item stored in BIMS and separates personally identifiable information from clinical and scientific data. Consolidation transforms imported data to a unified format [52]. Abstraction supports data presentation and control when researchers/users access the data through queries. The web-based query interface allows access for retrieval of data from the database [52]. 

Figure 2 illustrates an integrated knowledge environment through which personalized medicine can be approached. Within this environment, clinicians can determine if a patient has cancer and what course of treatment should be taken. Biomarker analysis will provide the information necessary to determine whether the patient should receive a conventional or customized treatment plan. For example, as noted earlier, the small fraction of NSCLC patients carrying a chromosome rearrangement resulting from an ALK mutation will have a poor outcome with conventional therapy but will do well with a personalized treatment that includes Xalkori, which is ineffective in a majority of NSCLS patients. A focus on sample quality is important in order to support “-omics” data and technologies with an eye towards clinical trials [53]. All experiments are dependent on the procurement and availability of high-quality, viable tissue specimens; thus, from the first step of procurement, it is crucial for the sample to be properly handled [54]. 

Collection, storage, and distribution of high quality tissue and blood samples require information supplied by bioinformatics data in concert with a reliable EMR system to link clinical data of interest. A significant key to proper tissue banking involves proper acquisition of samples by pathologists, who are responsible for determining what should be stored in the tissue bank by identifying the sample nature and origin, which makes pathology central to communication between scientists and clinicians [53]. As in bioinformatics, tissue banking requires collaborative efforts between academia, health institutions, and pharmaceutical companies. 

There are many issues, however, that pertain to tissue banking, including the loss of samples due to traceability and documentation errors and the need for coordination between multiple disciplines during the entire procedure. The Cancer Genome Atlas Project (an NCI initiative) assessed the quality of samples acquired from dozens of tissue banks and produced the surprising result that only one percent of the samples assessed were viable. In addition, most of the tissue banks that supplied these samples had no proper catalog of samples that were stored in their facilities [55, 56]. 

Well-established labs that have implemented a precise bank setup can maximize their research potential and, eventually, contribute to affordable health care diagnostics for patients as well as clinicians. Biobanks such as the UK Biobank, Biobanking and Biomolecular Resources Research Infrastructure (BBMRI), and Biobank Japan are among those that have created networks of organizations that manage millions of patient samples. Stored samples can be used to analyze cancers at various stages down to a molecular level. Because of the availability of samples from these biobanks, even for rare cancers, there would be no delay for sample collection since samples would already be available for experimentation [57]. These samples can be used for mechanistic studies for investigating the role of biomarker in disease progression or used with clinical data points and records (EMRs) to predict treatment options, drug responses, and susceptibility [58, 59]. By analyzing tumor samples and applying a comprehensive approach to determining the proper therapeutic regimen [60], each patient will have a better chance of a good outcome. However, if a given sample lacks relevant clinical data from the donor, experimental results will be more difficult to translate into real-practice medicine [55].

Samples available from tissue banks are used for experiments to discover and validate biomarkers to assist in translational research, identify diagnostic and prognostic targets to maximize patient benefit from customized care, and contribute to future diagnosis and treatment options [49]. The discovery, validation, and implementation phases of research on biomarkers require the resources found in tissue banks. Completion of the process and confirmation of biomarkers will lead to low-cost, yet efficient, and reliable medical care that will result in better patient care [49]. 

## 3. The Need for Electronic Medical Records (EMRs)

EMRs are used to improve patient care, and to date, their utilization has established high-quality practice-based datasets that are well suited for scientific research [61, 62]. A universal, user friendly, “Google-like” electronic medical record (EMR) system that allows crosstalk between various infrastructures is needed, since novel biomarker discovery is not possible unless clinical data are linked with patient samples that can be associated with clinical outcome [63]. To support this type of initiatives at national level, NCI has developed an electronicprogram providing a collection of cancer-related information, known as caBIG, and made it available to several hospitals and institutions. The sole purpose of the caBIG program is to assemble EMR files digitally in order for clinicians and scientists to facilitate translational medicine. However, caBIG function requires that an EMR setup be in place, and since many institutions have not yet instituted a proper EMR system, caBIG implementation has been slow. 

Early adopters of EMR technologies include Sweden and Denmark in the EU and Kaiser Permanente and The Veterans Health Administration in the USA. Even at early stage of EMR development, the success of IT implementations by these organizations is shown in their increased effectiveness in managing clinical data in electronic format. However, failed EMR projects in numerous organizations indicate that the currently implemented system needs to be revised. The major issues related to IT implementations are the complexity of managing the infrastructure, maintenance of alignment between organizations with different governance, managing funding, ensuring involvement of IT staff and managers, engagement of vendors, and adapting to changes to improve working methods [64]. 

In the USA, development of EMR will permit integration of biological data, clinical information, patient information, and clinical outcomes. Large populations or specific groups of patients with selected characteristics could be easily identified with the availability of electronic medical records. EMR can provide a larger number of participants, a wide range of information, and lower research costs [65]. In genomic research, EMRs facilitate analysis of genetic and molecular information from large subject populations allowing studies to be more powerful than smaller cohort studies. Even though there are difficulties associated with using information provided from EMRs, the lower cost of research and faster pace of advancement in clinical care help in overcoming the difficulties. In the USA, larger EMR databases are being linked to biospecimens procured from patients/donors covering a wide range of diseases. Analysis of biospecimens resulting in identification of genetic variants or Single Nucleotide Polymorphisms (SNPs) can be correlated and linked with available EHR information regarding parameters such as smoking history, obesity, cardio vascular diseases, and hypothyroidism [65]. 

A recent study using a descriptive, qualitative approach for exploring the experiences of primary healthcare providers who use EHRs in their ongoing practice identified factors that support and hinder the use of EHRs in healthcare. Factors that support the use of EHRs include improvement in efficiency of patient care and confidence in IT systems and software, whereas factors that hinder the use of EHRs include IT challenges related to computer usage such as scanning, electronic connectivity, and attaining proficiency in computer use. Generally, these factors that hinder use of EMRs can be addressed through training to enhance computer skills, along with consistent use of EMRs and data entry [66].

To apply innovative approaches in science and to produce the vast amounts of data required for biomarker analysis, it is essential to incorporate clinical data, which can only be made available through EMRs [67]. Translating scientific discoveries into medical practice is the most challenging part of personalized medicine, but once the scientific information (i.e., biomarkers, mutations, pathways, and drugs) is integrated with clinical data (i.e., survival, relapse, pathology, medications/treatments, and response), the translational power of bioinformatics will be apparent [68]. Other EMR advantages include communication between clinicians, scientists, and other health care providers, as well as notification of health care practitioners of common chemotherapy-related errors.

However, there are certain conflicts regarding the ethical issues related to EMRs. Exposure of health records by mistake, lack of security of system, and stealing can breach the confidentiality and fidelity of EMRs which might affect the treatment of patients [68, 69]. The health personnel who create the EMRs should be well aware of these issues. The Affordable Care Act will eventually require EMRs in every medical institution. EMRs require great attention to the challenges of electronically documenting care, the cost of building a large-scale EMR system to replace paper notes, and the cost of training employees [70]. When a universal EMR is in place, it will require management in structured data security, data recovery, and infrastructure inspection, which will be a general requirement for all medical institutions [71]. Insufficient medical information technology (IT) infrastructure and a lack of digitized clinical data are other issues that will almost certainly arise. Cancer care is complex, and to facilitate management of oncology patient records and workflow, detail-intensive data need to be organized in EMRs. Many cancer therapies involve substantial patient illness, and EMRs are an important clinical tool to improve patient safety, suitability, and efficiency, as well as patient-centeredness [72].

## 4. The Use of Bioinformatics and Integrated Knowledge Environments

Bioinformatics can enable clinicians to answer a fundamental question: based on all that is known about an individual patient, including disease characteristics, lab results, genomic, proteomic, and metabolomic information, what are the similarities to other patients who had good outcomes, and what is the best therapy for this patient with this disease? The progression of biomarker discovery is impossible without bioinformatics, which connects individual discovery processes, including experimental design, study execution, and bioanalytic analysis. Bioinformatics has dramatically progressed in the past decade with more than one million published articles in oncology research available in PubMed [42]. In the sphere of bioinformatics, large amounts of pertinent “-omics” data and information from the human genome are available for oncologic approaches to develop biomarker-related therapies. The NCI bioinformatics infrastructure caBIG makes available information from multiple studies to support new scientific efforts. The infrastructure also contains details of experiments, protocols, samples used, and specific results, which can be easily searched, compared, and downloaded from the database. NCI's Early Detection Research Network (EDRN) developed this program so that cancer institutions would have the bioinformatics data needed to support their research [73]. 

The EDRN exists today because of improvements in oncology research in the past decade that have derived from progress in bioinformatics. The direction from which studies are approached has been revolutionized by the essential usefulness of bioinformatic analysis of large-scale experimental datasets. Nonetheless, many cancer researchers are not accustomed to using bioinformatics and have not incorporated these advantages into their research. This will likely change as researchers grasp more fully the significance of the overlap between many areas of cancer research and bioinformatics with respect to data analysis and interpretation [74].

Translational research has been supported by bioinformatics, which has provided critical tools for transforming data into medical practice and has prompted biomarker breakthroughs and drug development (Figure 3). The use of clinically validated biomarkers will also permit development of cheaper, less invasive tests that will benefit both clinicians and patients [75]. However, the entire process will fail without the availability of properly curated patient clinical information with which to correlate the biomarker data. Bioinformatic results cannot be interpreted in the absence of clinical data and patient history, including treatment, physician's notes, pathology reports, and signs and symptoms [76]. The failure to implement use of EMRs is a critical aspect that delays integration of patient records with experimental results, yet many healthcare facilities still rely on paper notes [77]. Often, hospital personnel, including clinicians, lack the training, interest, or time to learn and understand the benefits of bioinformatics. There is no mystery to this, health care providers are occupied with patient care, and there is no reimbursement for entering clinical data into a database. Nonetheless, the participation of clinical personnel in bioinformatics research is vital and must be encouraged. 

Patient EMR information, including demographics and disease characteristics, combined with tissue bank-derived experimental results, can be used to create a personalized patient genomic profile or “information object” that can allow clinicians to query integrated bioinformatics systems to compare each individual patient with current and historical information. The results from mining and comparing patient profiles can produce an “intelligent” picture of the therapies and treatments that have the highest probabilities for the best outcomes. Systems to support clinicians must be fast, easy to access, and must provide clear answers to personalized medicine questions. 

**Figure 1:**
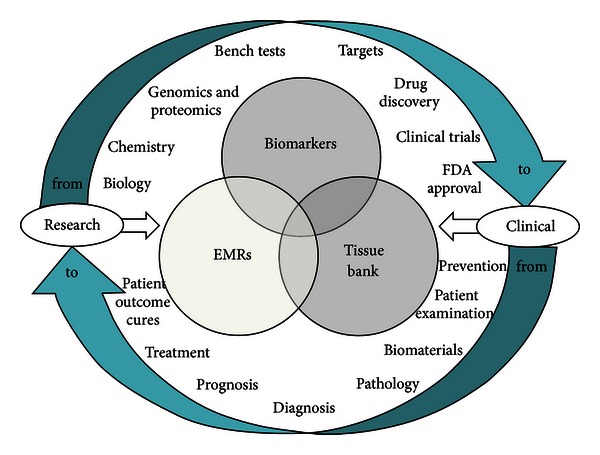
The Sophic Systems Alliance Inc. diagram shows the integrated “knowledge environment” that enables clinicians to query critical information from across disparate data sources to find relationships between an individual patient's EMR information.

**Figure 2:**
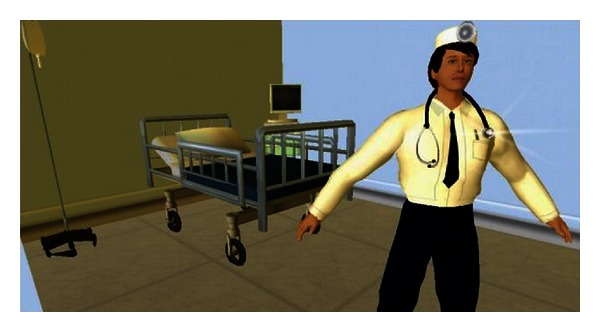
Scenario of a virtual platform that can demonstrate how patients are asked to donate tissue and/or blood samples in real-world situations with a game-like characteristic.

**Figure 3:**
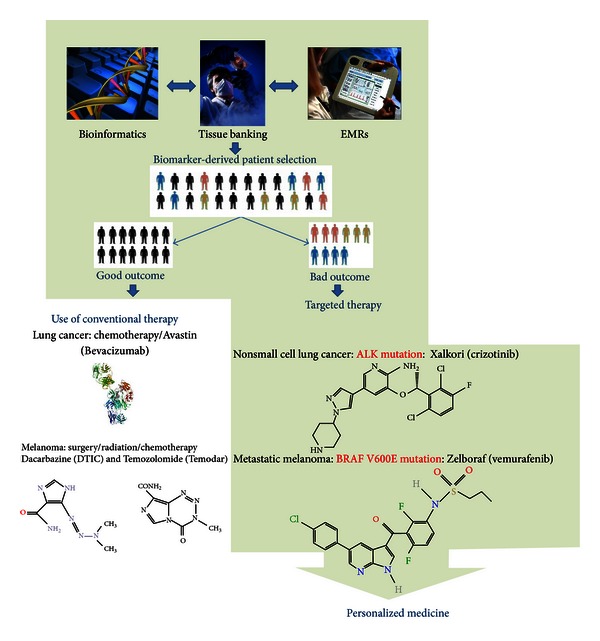
Based on the data resulting from the combination of bioinformatics, tissue banking and EMRs, novel biomarkers can predict if a patient will go through the normal conventional therapy or require a personalized treatment plan based on the type of mutation and cancer is present.

To identify optimal therapies for each unique circumstance, clinicians need easy access to “intelligence”, which is provided through the integration and correlation of critical information stored in disparate and constantly changing data sources. Successful integration requires a three-way alignment of information stored in the patient's EMR, tissue bank pathology test results, and genomic information. Clinical data on patients, diseases, therapies, and outcomes are stored in hospital patient databases and public sources such as NCI's TCGA and TARGET databases. To advance the cause of personalized medicine, these clinical databases should allow easy access to outcomes data for each patient cohort for specific diseases and associated therapies, which, along with related drug safety and toxicity information (FDA Drug Labels, DrugBank), is critical to decision making regarding the care of each patient (Figure 1).

Kaplan-Meier chart information can also allow clinicians to quickly and easily review details, sources, and specifics of evolving information to gain the confidence required to decide on a specific treatment regimen. Bioinformatic support for clinicians should integrate the ability to produce Kaplan-Meier charts for easy reference. The development of bioinformatics to support personalized medicine has been underway in government, academic, and private hospital research cancer centers for years. Progress is being made toward resolving some of the more complex obstacles for translating and integrating disparate data sources. Although the current state of bioinformatics is imperfect, collaborations between government, academic, and biotech and pharmaceutical companies have produced advances in standards, ontologies, and vocabularies that support integration of critical information. Currently available flexible data models provide scientific, medical, and semantic relationships between critical data elements that are often stored in various systems and databases. The virtual integration provided by data models enables querying, mining, searching, retrieving, and accessing critical information necessary to support accurate decision making. 

Integration is achieved through community-wide initiatives. HL7 standards enable translation and communication of patient information stored in EMRs, while Gene Ontology and NCI's Thesaurus help align vocabularies and terms across research databases. Clinical and biological databases such as those hosted by the NCBI as well as others such as Reactome and Sanger's COSMIC mutation database provide evolving and improving reference databases from which information can be mined and aligned with laboratory data, sequence information, and genetic mutations to help clinicians decide on treatment options with the highest likelihood of positive outcomes.

The synthesis and analysis of information from these various sources can expose relationships between critical data elements that provide “intelligence” and can identify the best therapeutic course to deliver the best outcome. Academic cancer treatment hospitals (e.g., MD Anderson, Mayo Clinic) have led the way in developing software and technologies that allow data integration and EMR mining. Commercial software companies are delivering innovative “knowledge environments” such as Sophic's SCan-MarK Explorer cancer biomarker database, research, mining, and discovery system, which is built on Biomax BioXM technology. With software like BioXM, experimental designs can focus on predictive biomarkers that indicate whether a customized treatment plan is needed for certain patients. In biomarker research, scientists examine how biomarkers are formed, how they function, and how that function relates to patient data [75]. Before biomarkers can be adopted for practical use in the format of assays and for detection by various biosensors, patient information and bioinformatics need to be combined to generate hypotheses that can be tested using clinical samples stored in tissue banks [76]. Bioinformatics, in particular, provides an irreplaceable infrastructure to support accompanying multidisciplinary education and research [77].

In the future, bioinformatics will enable the customization of medical care to the specific genome of each patient rather than providing a single, conventional treatment [78]. There will be an abundance of information that can only be approached through bioinformatic analysis [79]. Cancer is complicated, and biomarkers promise advancement of early diagnosis and targeted therapies, which would not be possible without bioinformatics tools. In the case of Hodgkin's lymphoma, up to 20% of patients will relapse after receiving conventional frontline therapy. Without bioinformatic data analysis to design clinical studies and query “-omics” information, these “fingerprints” of cancer would be difficult or impossible to find [80]. The strategy that should be adopted is to take potential biomarkers and use them to determine the diagnosis, prognosis, and course of therapy for specific cancers. Biomarker discoveries have the capability of improving early diagnosis, as well as guiding customized therapies and enhanced monitoring after treatment [81]. Thus, the effective use of bioinformatics in clinical setting by analyzing clinical data from EMR along with biomarker-derived diagnostic or prognostic assays can provide clinical decision making steps that could be of long-term benefit to patients [80].

## 5. Conclusions

Health care practitioners need more training in the areas of bioinformatics and tissue banking, and EMR systems need to be in place to provide the necessary components for biomarker discovery that can lead to customized medical care for cancer patients. Training can be accomplished by (i) presenting bioinformatics as a helpful toolkit; (ii) promoting improved training of individuals in proper protocols for tissue procurement and storage; (iii) encouraging documentation of all patient information using EMRs instead of paper charts; and (iv) fostering further collaborative efforts between clinicians and scientists. Methods for providing more information about the benefits of tissue sample donations and increasing informed consent can be improved to ensure richer data sources for determining patterns and personal differences. With these enhancements in place, the focus can be shifted to the discovery of predictive, diagnostic, and prognostic biomarkers that will allow proper diagnosis of specific cancers, increase information about a particular disease, and indicate the direction treatment should take. Community-wide efforts for collecting patient information, creation of well-controlled tissue banks, and development of new genomic and proteomic technologies will provide clinicians with the building blocks for achieving personalized medicine and improving patient treatment outcomes. These efforts will also provide patients with reduced medical care costs. On the long run, adoption and integration of bioinformatic tools into routine clinical use will save lives.

## Figures and Tables

**Table 1:** List of biomarkers and personalized medicines with companion diagnostics.

Biomarker	Cancer type (subtype)	Companion diagnostics (company)	Drug therapy (company)	Reference
HER2 (gene amplification)	Breast cancer (HER 2 positive)	**SPoT-Light HER2 CISH **Hercep test (Life Technologies, NY)	**Herceptin, trastuzumab (Hoffman La Roche Inc.)	[13, 14]

ALK (chromosome rearrangement)	Nonsmall cell lung cancer (anaplastic lymphoma kinase (ALK) positive advanced nonsmall cell lung cancer)	**Vysis ALK FISH test (Abbott Laboratories, Abbott, IL)	**Xalkori, crizotinib (Pfizer)	[13, 15]

EGFR KRAS (mutation)	Colorectal Cancer (expressing metastatic colorectal carcinoma, EGFR)	**Therascreen KRAS Test (Qiagen, Corporate Headquarters, Nederland's)	**Erbitux, cetuximab (ImClone, ImClone Systems, NJ) **Vectibix, panitumumab (Amgen, Amgen Inc,. CA)	[13, 16, 17]

BRAF V600E (mutation)	Melanoma (metastatic melanoma with BRAFV600E mutation)	**Cobas 4800 BRAF V600 Mutation Test (Panagene, Corporate Headquarters, Korea)	**Zelboraf, vemurafenib (Genentech/Roche)	[13, 18]

BRCA1/2 (gene translocation)	Breast cancer (median, triple-negative, HER2+, and ER+/HER2−)	N/A	*Veliparib, ABT-888 (Abbott) *Olaparib, AZD2281 (Abbott)	[13, 19, 20]

PML-RAR (gene translocation)	Acute Promyelocytic Leukemia	N/A	**Trisenox, arsenic trioxide (Teva, Israel)	[13, 23]

BCR-ABL (gene translocation)	Chronic myelogenous leukemia	N/A	**Gleevec, imatinib (Novartis) **Sprycel, dasatinib (Bristol Myers Squibb) **Tasigna, nilotinib (Novartis)	[13, 24, 25]

C kit, FIP1L1-PDGFR*α*	Chronic myeloid leukemia (Ph+ CML) gastrointestinal stromal tumor (GIST)	**DAKO C-KIT PharmDx	**Dako North America, Inc.	[26]

CD 20	Non-Hodgkins lymphoma (CD20+ follicular B-cell non-Hodgkin's lymphoma)	*Rituxan Sensitivity (CD20), Flow cytometry assay	**Bexxar, tositumomab (GlaxoSmithKine)	[27]

CD 25	T-cell lymphoma (cutaneous T-cell lymphoma,)	*ONTAK Sensitivity (CD25), Flow Cytometry (Quest Diagnostics)	**Ontak, denileukin diftitox (Marathon Biopharmaceuticals Inc. MA)	[28]

CD 30	Refractory Hodgkins lymphoma	*Fluorescent microsphere immunoassay, (Quest Diagnostics)	**Adcetris, brentuximab vedotin (Seattle genetics Inc.) Corporate Headquarters, Seattle Genetics, Inc., WA, USA.	[29]

TPMT	(CD30+ lymphoma)	*TPMT Activity, Liquid Chromatography Tandem Mass Spectrometry (LC/MS/MS) (Quest Diagnostics)	**Tabloid, thioguanine (GlaxoSmithKine)	[30]

DPD	Breast cancer (with TS, MTHFR, and DPD gene polymorphisms)	*Polymerase Chain Reaction (PCR) Single Nucleotide Primer Extension	**Xeloda, capecitabine (Hoffman La Roche Inc.)	[31]

ER-PGR	Breast cancer (ER and/or PGR+)	*Immunohistochemistry (IHC) (Quest Diagnostics)	**Aromasin, exemestane (Pfizer)	[32]

G6PD	Lymphoma, leukemia (lymphoid leukemia (B and T cell), non-Hodgkin's lymphoma (including Burkitt's lymphoma) or acute myelogenous leukemia)	N/A	**Elitek, rasburicase (Sanofi-synthelabo Inc.)	[33]

ER	Breast cancer (low-grade ER−/PR+)	*Immunohistochemical Assay (Quest Diagnostics)	*Nolvadex, tamoxifen (AstraZeneca)	[34]

ER	Breast cancer (ER+)	N/A	*Arimidex, anastrozole (AstraZeneca)	[35]

ER	Breast cancer (ER+ and/or PgR+)	N/A	**Faslodex, fulvestrant (AstraZeneca)	[36]

UGT1A1	Colorectal cancer (UGT1A1*28 polymorphisms)	*Fluorescent polymerase chain reaction (PCR) with primers specific for the 5′ untranslated region of UGT1A1 (Third Wave Technologies, WI)	**Camptosar, irinotecan (Pfizer)	[37]

ERCC1	Gastrooesophageal cancer (with ERCC1 nuclear protein expression)	N/A	*Camptosar, irinotecan (Pfizer)	[38]

TPMT	Metastatic Testicular tumors Metastatic ovarian tumors Advanced bladder cancer	N/A	*Platinol, cisplatin, (Bristol-Myers Squibb Company)	[39]

TPMT	Acute nonlymphocytic leukemias	N/A	**Tabloid, thioguanine (GlaxoSmithKline)	[40]

CDK 4 and 6	Breast cancer (luminal estrogen receptor-ER+, HER2−)	N/A	*Palbociclib, PD-0332991 (Pfizer)	[41]

*This biomarkers are used for diagnostic and prognostic purposes to determine the proper cause of treatment (investigational diagnostics and/or drugs).

**FDA approved diagnostics and/or drugs.
